# Quantification of Uncoupling Protein 2 Reveals Its Main Expression in Immune Cells and Selective Up-Regulation during T-Cell Proliferation

**DOI:** 10.1371/journal.pone.0041406

**Published:** 2012-08-03

**Authors:** Anne Rupprecht, Anja U. Bräuer, Alina Smorodchenko, Justus Goyn, Karolina E. Hilse, Irina G. Shabalina, Carmen Infante-Duarte, Elena E. Pohl

**Affiliations:** 1 Institute of Physiology, Pathophysiology and Biophysics, University of Veterinary Medicine, Vienna, Austria; 2 Institute of Cell Biology and Neurobiology, Charité – Universitätsmedizin, Berlin, Germany; 3 The Wenner-Gren Institute, Stockholm University, Stockholm, Sweden; 4 Experimental and Clinical Research Center, a joint cooperation between the Charité - Universitätsmedizin Berlin and the Max-Delbrück Center for Molecular Medicine, Berlin, Germany; Center of Ophtalmology, Germany

## Abstract

Uncoupling protein 2 (UCP2) is an inner mitochondrial membrane protein. Although the protein was discovered in 1997, its function and even its tissue distribution are still under debate. Here we present a quantitative analysis of mRNA and protein expression in various mice tissues, revealing that UCP2 is mainly expressed in organs and cells associated with the immune system. Although the UCP2 gene is present in the brain, as demonstrated using quantitative RT-PCR, the protein was not detectable in neurons under physiological conditions. Instead, we could detect UCP2 in microglia, which act in the immune defense of the central nervous system. In lymphocytes, activation led to a ten-fold increase of UCP2 protein expression simultaneously to the increase in levels of other mitochondrial proteins, whereas lymphocyte re-stimulation resulted in the selective increase of UCP2. The highest detected level of UCP2 expression in stimulated T-cells (0.54 ng/(µg total cellular protein)) was approximately 200 times lower than the level of UCP1 in brown adipose tissue from room temperature acclimated mice. Both the UCP2 expression pattern and the time course of up-regulation in stimulated T-cells imply UCP2’s involvement in the immune response, probably by controlling the metabolism during cell proliferation.

## Introduction

Uncoupling protein 2 (UCP2) is an inner mitochondrial membrane protein, which belongs to the mitochondrial anion carrier superfamily [1], [2] and is highly homologous to UCP3 (73%) and UCP1 (59%, [3]). The latter mediates non-shivering thermogenesis in brown adipose tissue (BAT) by dissipating a proton gradient across the inner mitochondrial membrane [4]–[6]. Although UCP2 was discovered in 1997, its transport function is still under debate. Experiments using artificial membranes support the idea that UCP2 transports protons in the presence of fatty acids and with high sensitivity to the membrane potential similarly to UCP1 [7]–[10]. However, there is still no convincing evidence from cells or isolated mitochondria that the uncoupling properties of UCP2 can be compared to those of UCP1. A possible explanation may be the difference in the expression levels of these proteins [11].

Unlikely to UCP1 and other UCPs, UCP2 mRNA was detected in all tissues tested [3], [12]–[15]. In contrast to mRNA, protein expression has been described in few tissues and tissue distribution reported by different laboratories varies. One group reported the UCP2 expression in spleen, lungs, stomach, intestine, white adipose tissue (WAT) but not in muscle, heart, liver, kidney, BAT and brain [16], [17], while other laboratories have found UCP2 in pancreatic islet cells [18], thymocytes [19] and kidney [20]. Turner et al. [21] detected UCP2 in cardiomyocytes. Several research groups revealed the presence of UCP2 in distinct brain regions [22]–[24]. Possible reason for the discrepancies are the different animal models used, the age of the animals, the quality of the antibodies used and the extremely short half-life time of the protein [25].

Despite the fact that UCP2 expression in brain has not been clearly demonstrated, UCP2 is increasingly associated with a neuroprotective function [26]–[31]. Sometimes such conclusion has been supported by artificial overexpression of UCP2 [32] that may not correctly reflect normal tissue distribution. Many previous observations were done using UCP2 knockout mice on mixed genetic background [18], [33]. An increase of UCP2 mRNA levels in the course of mice experimental autoimmune encephalomyelitis [34] was reported, however, it could not be determined whether UCP2 is increased due to spinal cord infiltration by immune cells or as a result of neuronal expression of UCP2.

The first functional connection between UCP2 and immune system was established when the UCP2 knockout mouse was found to show an elevated immune response to pathogens [33]. An increasing number of reports demonstrate that macrophages and mast cells from the UCP2 knockout mouse have higher levels of cytokines or histamine, are more active by infiltration and produce more reactive oxygen species (ROS) [35]–[38]. The analysis of UCP2 levels after macrophages activation [39] revealed that UCP2 expression is reduced after stimulation with lipopolysaccharide (LPS) for 24 hours. No results concerning UCP2 regulation at later time points after stimulation were reported. In contrast, in vivo studies showed up-regulation of UCP2 after LPS treatment, explaining the discrepancy between in vitro and in vivo studies by existing of two, early and late, stages of the immune cell response [40]. It is possible that other cells beside immune cells contribute to the UCP2 expression in the studies in vivo. The regulation of the UCP2 expression in stimulated T-cells is still not investigated. It is also unclear, whether UCP2 abundance varies selectively or simultaneously to other mitochondrial proteins.

In the present work we investigate UCP2 expression among different mouse tissues at the mRNA and protein levels focusing on cells of the nervous and immune systems. For this, we generated new antibodies that had given the correct results with recombinant UCP2 and the UCP2 knockout mouse. To test the hypothesis that UCP2 up-regulation in brain during inflammation may be explained by enhanced protein expression in immunocompetent cells (housing and/or invaded) rather than in neurons, we compared the expression of UCP2 at different activation stages of lymphocytes.

## Methods

### Chemicals

All chemicals were obtained from Sigma-Aldrich (Austria), Merck (Austria) or Lactan (Austria), unless otherwise indicated.

### Animals and Tissue Samples

Native C57BL/6 mice aged 5 days, 30 days, 5 months and 12 months were purchased from Charité – Universitätsmedizin’s central animal facility (FEM) (Berlin, Germany) or Charles River (Sulzfeld, Germany). Animals were deeply anaesthetised with a mixture containing ketamine (Pfizer, Germany) and xylazine (Rompun@, Bayer, Germany) and sacrificed by decapitation as previously described [41]. Brain, spinal cord, thymus, heart, testis, white adipose tissue, skeletal muscles, lung, spleen, lymph nodes, stomach, intestine, liver, and kidney were removed, frozen in liquid nitrogen and stored at −80°C until protein or RNA isolation was performed. All procedures in this study were performed in accordance with the European Communities Council Directive from November 24th, 1986 (86/609/EEC).

Male C57Bl/6 mice lacking UCP2 (UCP2^−/−^) and their wild type controls aged 10–12 weeks were derived from those described in [33]. The UCP2^−/−^ mice used in this study were backcrossed for more than 10 generations onto a C57Bl/6 background. Animals were maintained on a 12∶12 h light-dark cycle (light from 7 a.m. to 7 p.m.), at 24°C and were allowed unlimited access to standard laboratory chow and tap water. All experiments were approved by the Animal Ethics Committee of the North Stockholm region.

### Magnetic Immune Cell Separation

Mononuclear splenocytes were isolated on Histopaque gradient from three independent native C57BL/6 mice. Monocytes, T-cells and B cells from C57BL/6 mouse spleen were positively selected by magnetic cell separation using MACS-beads (Miltenyi Biotec, Germany) coated with antibodies against CD11b, CD4 and CD19, respectively. NK cells were negatively selected using NK cell isolation kits (Miltenyi Biotec, Germany). Cell populations were sorted according to the manufacturer’s instructions and yielded >95% purity. After sorting, washing and counting, immune cells were processed immediately for protein extraction as described previously [41].

### Primary Brain Cell Cultures

Cortical neuron, astrocyte and microglia cultures were prepared as previously described [41]. For RNA and protein isolation, neurons were harvested after 11–12 days, astrocytes and microglia after 13–21 days.

### Isolation and Activation of OT-II T-cells

We used naive T-cell-receptor transgenic T-cells (background C57BL/6) specific for the ovalbumin peptide OVA323–339 (OT-II T-cells) because of their clonal nature and specificity to an exogenous antigen. OT-II T-cells were magnetically sorted using anti-CD4-MACS beads according to the manufacturer’s instructions (Miltenyi Biotec, Germany) and primed in vitro with OVA323–339 (Pepceuticals, UK) presented by sorted irradiated antigen-presenting cells (APC) at a ratio of 1∶10 (T-cells: APC). T-cells and APC were cultured in 3 × 10^6^/ml cell culture medium (RPMI 1640 supplemented with 2 mM L-glutamine, 100 U/ml penicillin, 100 µg/ml streptomycin and 10% foetal calf serum) for 7 days. Thereafter, viable OT-II cells were isolated by density gradient centrifugation with Histopaque-1077 (Sigma-Aldrich, USA). T-lymphocytes were re-stimulated at day 7 after the first stimulation for the maintaining of high cell activity. On day 3 and 5, T-cells were splitted with fresh medium containing IL-2 (100 U/ml). To avoid the presence of APC-derived proteins in Western blot analyses, OT-II cells were re-stimulated without APC using coated anti-CD3 and anti-CD28 antibodies (BD Biosciences, Germany) and analysed after 6, 24 hours and 2, 3, 4, 7, 10 and 14 days. The second re-stimulation was performed identically 7 days after the first re-stimulation. After various periods of activation, T-cells were processed for protein extraction as described below. In cultures, in which expression analyses were performed from day 0, OT-II T-cells were activated with aCD3/aCD28 antibodies from the first day on in order to avoid APC contamination in the Western blot analyses as mentioned above.

### Isolation and Activation of Wild Type CD4 and CD8 T-cells

CD4 and CD8 T-cells were magnetically sorted from C57BL/6 splenocytes using anti-CD4 and anti-CD8 MACS beads, respectively according to the manufacturer’s instructions (Miltenyi Biotec, Germany). CD4 and CD8 T-cells were primed in vitro with plate-bound aCD3/aCD28 antibodies and analysed after 6, 24 hours and 2, 3, 4, and 7 days as described above.

To rule out potential effects of the culturing medium containing glutamine and IL-2 on the expression of UCP2, CD4 T-cells were cultured in the absence of aCD3/aCD28 antibodies with IL-2 (100 U/ml) added on day 0 h and at 72 h using standard medium, i.e. RPMI 1640 supplemented with 2 mM L-glutamine and antibiotics. Expression of UCP2 was monitored at 24 hours and on day 2, 3 and 4 after stimulation (Fig. S1C).

### Quantitative RT-PCR

Mouse organs obtained from six independent animals for each of the investigated ages were snap-frozen immediately after extraction in liquid nitrogen. Mouse tissues and primary mouse brain cells (neurons, astrocytes and microglia) were homogenized in TRIzol reagent (Invitrogen). Total RNA was purified according the TRIzol protocol. RNA concentrations were determined at wavelength 260 nm using a Biomate 3 Spectrometer (Fisher Scientific, USA). A “High Capacity cDNA Reverse Transcription kit” (Applied Biosystems, USA) was used to generate total cDNA for the real-time PCR using 5 µg total RNA from each sample according to the manufacturer’s recommendations.

Each PCR reaction contained: 8 µl H_2_O, 10 µl TaqMan® Fast Universal PCR Master Mix (Applied Biosystems, USA), 1 µl cDNA and 1 µl TaqMan Gene Expression Assays. GAPDH (glyceraldehyde-3-phosphate dehydrogenase), ß-actin and HRPT (hypoxanthine-guanine phosphoribosyltransferase) were used as internal controls. Assays ID 4352932E and ID 4352933E were used for GAPDH and β-Actin respectively. For HPRT separate primers and probes were used (Primer Mix (for 5′-ATCATTATGCCGAGGATTTGGAA-3′; rev 5′-TTGAGCACACAGAGGGCCA-3′) and probe (5′-TGGACAGGACTGAAAGACTTGCTCGAGATG-3′). The investigated TaqMan Gene Expression Assays was UCP2 (uncoupled protein 2, Assay ID 627598 m1). To determine the relative gene expression in each experiment samples were double-tested and one ‘no template’ control (NTC) was used. Each result is the average of three separate experiments. All transcripts (within each set of specimens) were always analysed within the same experiment. Amplification and fluorescence detection were conducted with the ABI PRISM™ 7700 Sequence Detection System (Applied Biosystems). The fluorescence threshold value was calculated using the ABI PRISM™ 7700 System Software.

For the quantitative comparison of the UCP2 gene expression, data extracted from each qRT-PCR run were analyzed using the 7500 Fast System software (Applied Biosystems, Foster City, CA, USA). The value of the noise fluorescence, usually indicated as the base line of the run, was automatically determined. The threshold value was manually set to 0.05. The CT was automatically calculated and used to quantify the starting copy number of the target mRNA. Normalization of UCP2 gene in tissue and cells was carried out to internal control of GAPDH, ß-actin and, HPRT expression by means of the 2-dCT method [42].

### Western Blot Analysis

The collection of total cellular protein from tissue samples and Western blot analysis was performed as described previously [41] with some modifications. Per lane 20–50 µg of protein were loaded. Membranes were incubated at 4°C in block solution (1x TBS-buffer; 2% BSA; 0.05% Tween 20; 0.02% Thimerosal) for at least 12 hours. Samples were incubated with UCP2 (s. Results) antibodies (diluted 1∶2000) in block solution for up to two days at 4°C. As secondary antibody, horseradish peroxidase-linked antibody (GE Healthcare, Austria) was used. Immunoreaction was visualized with luminescence from ECL Western Blotting reagent (GE Healthcare, Austria) used according manufacturer’s instructions and measured with the ChemiDoc-It 600 Imaging System (UVP, UK). For protein loading controls, membranes were incubated with specific antibodies against VDAC (Abcam, ab14734, dilution 1∶5000), Hsp60 (heat shock protein, Abcam, dilution 1∶10000), SDHA (succinate dehydrogenase complex, subunit A, Abcam, dilution 1∶10000), β-Actin (Sigma-Aldrich, A5441, dilution 1∶5000) or GAPDH (Sigma-Aldrich, G8795, dilution 1∶10000) in block solution for at least one hour.

## Results

### UCP2 mRNA Expression Pattern

**Figure 1:**
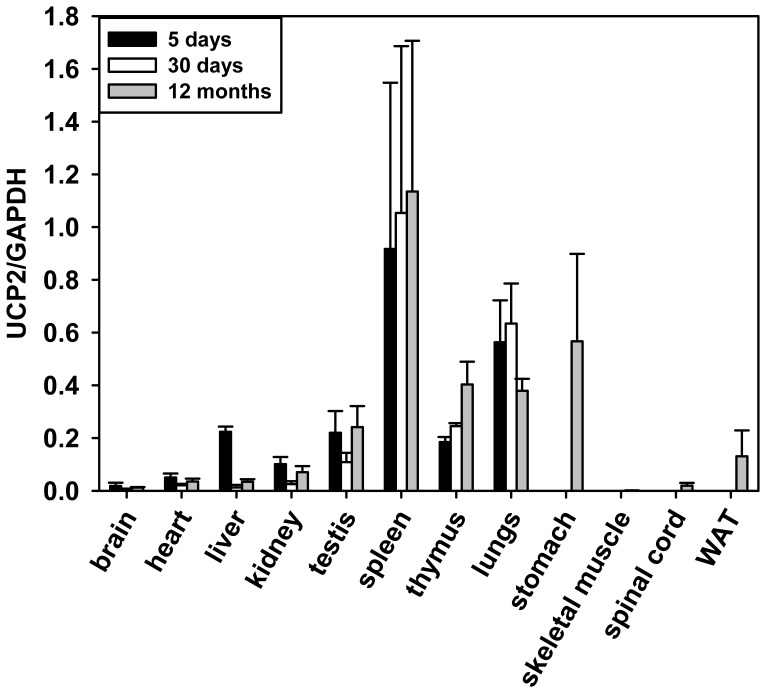
Quantitative analysis of UCP2 mRNA distribution in various tissues by real-time PCR in 5 day, 30 day and 12 month old mice. UCP2 mRNA levels are represented as ratios to the mRNA level of the housekeeping gene GAPDH. UCP2 mRNA in stomach, skeletal muscle, spinal cord and WAT was measured only in 12 month old mice. Each data point represents the mean value ± SD of 3–6 mice.

Although all previous studies have shown that UCP2 mRNA is ubiquitously distributed, the reported levels vary considerably between tissues. To test whether age differences may have contributed to the discrepancies in results, we compared UCP2 mRNA expression in different tissues of early postnatal (5 days old), young (30 days old) and adult (12 months old) C57BL/6 mice (Fig. 1). mRNA levels were determined by quantitative real-time PCR-analysis and presented as ratios of UCP2 to the housekeeping gene GAPDH. We detected the highest UCP2 mRNA expression in spleen with mRNA levels (1.13±0.57) times those of GADPH. Thymus, lung and stomach showed slightly lower mRNA levels at 0.40±0.09, 0.38±0.05 and 0.57±0.33, respectively. UCP2 mRNA levels determined in WAT and kidney were 0.13±0.10 and 0.07±0.02. The lowest UCP2 mRNA levels were measured in skeletal muscle (0.001±0.0005), whole brain (0.013±0.002) and spinal cord (0.02±0.01). Notably, UCP2 mRNA levels in most investigated tissues, including brain, heart, lungs, kidney and especially liver, are down-regulated in adult mice compared to early postnatal age. Exceptions are the spleen and thymus, in which we observed an increase of UCP2 mRNA levels with aging. Although some of our mRNA analysis is in agreement with the observation of other groups [14] no such tendency has been reported previously. UCP2 mRNA levels compared to β-actin show comparable results (data not shown).

### Antibody Generation and Validation

**Figure 2:**
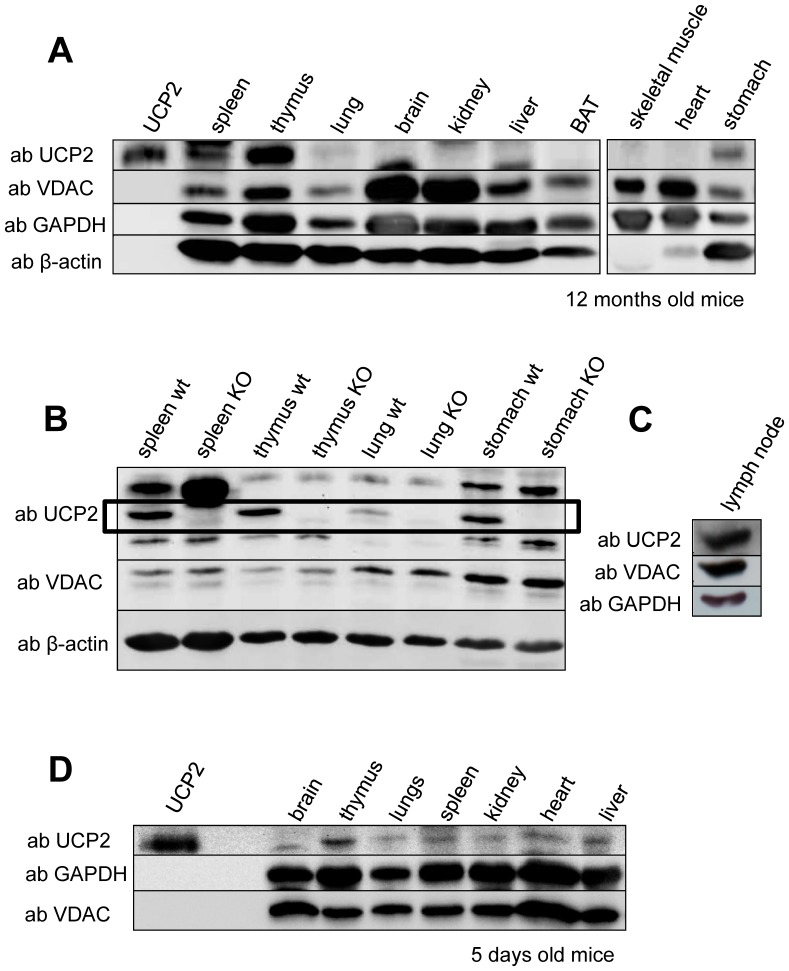
Representative Western blot analysis showing UCP2 expression in different tissues from 12 month old mice (A), UCP2 knockout mice and their wild type controls aged 10–12 weeks (B) and 5 day old mice (D). Analysis of UCP2 protein expression in lymph nodes from 12 week old mice (C). Gels were loaded with 50 µg protein per lane. Recombinant human UCP2 was used as a control. At least six mice were analysed at each condition.

To obtain specific antibodies against UCP2, two peptides, (1) “VGFKATDVPPTATVKF” (homologous to the mouse UCP2 N-terminus) and (2) “DSVKQFYTKGSEHAGIGSR” (homologous to the first innermembrane space loop of mouse UCP2) were synthesized. 180 days after rabbit immunization, serum was affinity purified and tested in Western Blots, using recombinant mUCP1 [10], hUCP2 [10], mUCP4-gfp, mUCP5 [41] and tissue samples from UCP2 knockout mice. Synthesis of peptides, immunization of rabbits and affinity purification of serum, containing UCP2 was performed by PINEDA Antibody-Service GmbH (Berlin, Germany). Only the antibody generated using the peptide “VGFKATDVPPTATVKF” recognized the recombinant UCP2 and showed no cross reaction with other recombinant proteins. No protein recognition was observed if tissues from the UCP2 knockout mouse were used (Fig. 2B).

### UCP2 Distribution at the Protein Level

To compare UCP2 mRNA and protein levels, we performed Western blot analysis using the same tissue samples. In thymus, spleen, lungs and stomach tissue of 12 month old mice we detected bands at the same position as that of recombinant UCP2 (Fig. 2A). We verified these bands by analysis of UCP2 knockout mouse tissues (Fig. 2B). Under the same conditions we detected UCP2 in intestine, colon (data not shown), but did not find bands corresponding to UCP2 in brain, liver, BAT, skeletal muscle or heart. The observed expression pattern matches the reported mRNA levels in these tissues and is generally in accordance with the analysis published in [16]. To test the hypothesis that UCP2 is abundant in all organs of the immune system, we additionally investigated lymph nodes (Fig. 2C), where we also detected a significant level of UCP2 expression. The expression in testis (data not shown), kidney and brain was rather sporadic (Fig. 2). We could not distinguish, whether UCP2 is expressed in functional cells of these organs or whether the signal we observed was caused by housing immune cells.

Whereas 30 days old mice showed a similar pattern of UCP2 protein expression in Western blot analysis as that of adult mice (data not shown), we detected an additional slight but stable protein expression in kidney, heart and liver (Fig. 2D) of early postnatal mice (5 days old). The expression in liver may be explained by the presence of erythroblasts due to foetal erythropoiesis [43]. No significant UCP2 expression in whole brain was found in any of the age groups examined.

### UCP2 Expression in Regions of the Central Nervous System

**Figure 3:**
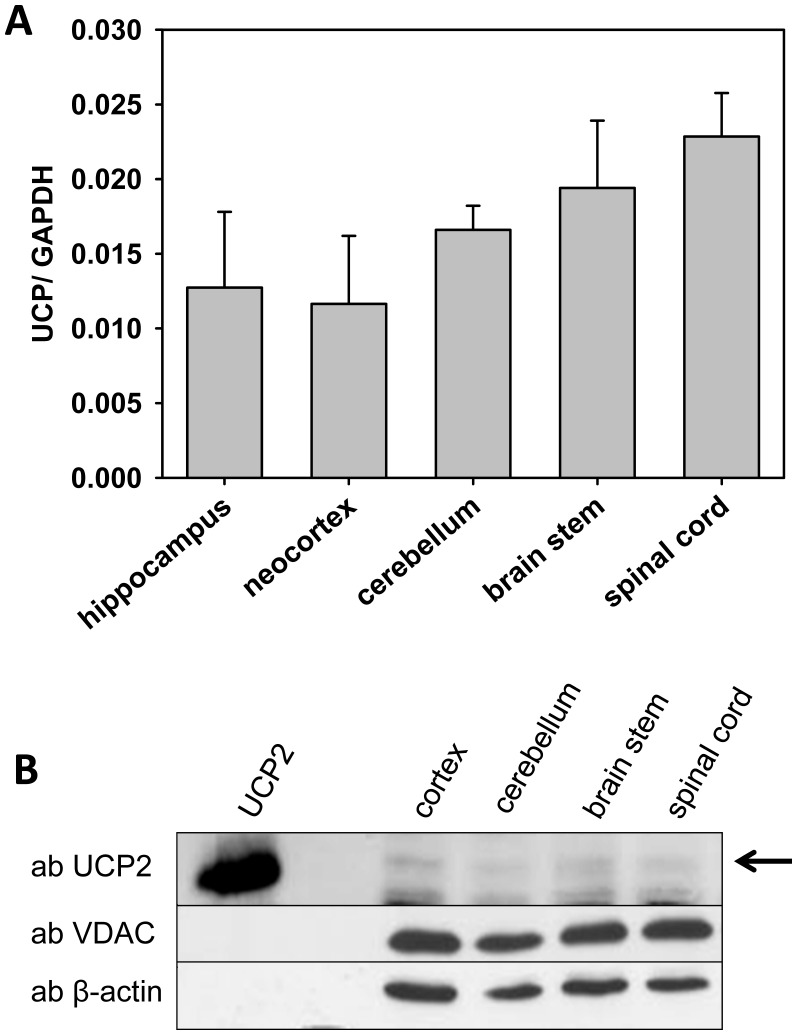
Comparison of UCP2 mRNA and protein levels in different regions of the central nervous system. (A) UCP2 mRNA determination in 5 month old mice. UCP2 values are presented as ratios to the amount of GAPDH mRNA. Each data point represents the mean ± SD of six mice. (B) The best example for UCP2 protein detection in 50 µg total cellular protein of various regions of the central nervous system was performed in 5 month old mice.

It was previously described that UCP2 expression in brain is restricted to particular regions, such as the supraoptic, paraventricular, suprachiasmatic and arcuate nuclei of the hypothalamus [22]. To test whether UCP2 expression is specific to these defined regions of the brain, we investigated the neocortex, hippocampus, cerebellum, brain stem and spinal cord for mRNA and protein expression. Figure 3A shows the distribution of UCP2 mRNA at similar levels in neocortex (0.012±0.005), hippocampus (0.013±0.005), cerebellum (0.017±0.002), brain stem (0.019±0.005) and spinal cord (0.023±0.003) related to the housekeeping genes GAPDH or HRPT (data not shown). Using Western Blots we detected no differences in the levels of UCP2 protein expression between cortex, cerebellum, brain stem and spinal cord. Only at prolonged developmental times for detection of chemiluminescence and with larger amounts of total protein applied (50–100 µg) did we occasionally obtain a barely visible signal for UCP2 protein expression in the regions tested. Figure 3B shows the best example for the Western Blot. Interestingly, although UCP2 mRNA was detectable (Fig. 3A), UCP2 protein level seems to be very low. Based on these data we hypothesized that the traces of UCP2 could be due to the presence of microglia rather than due to UCP2 expression in neuronal cells as previously reported at the mRNA level [26], [44].

### Analysis of UCP2 Expression in Brain Cells

**Figure 4:**
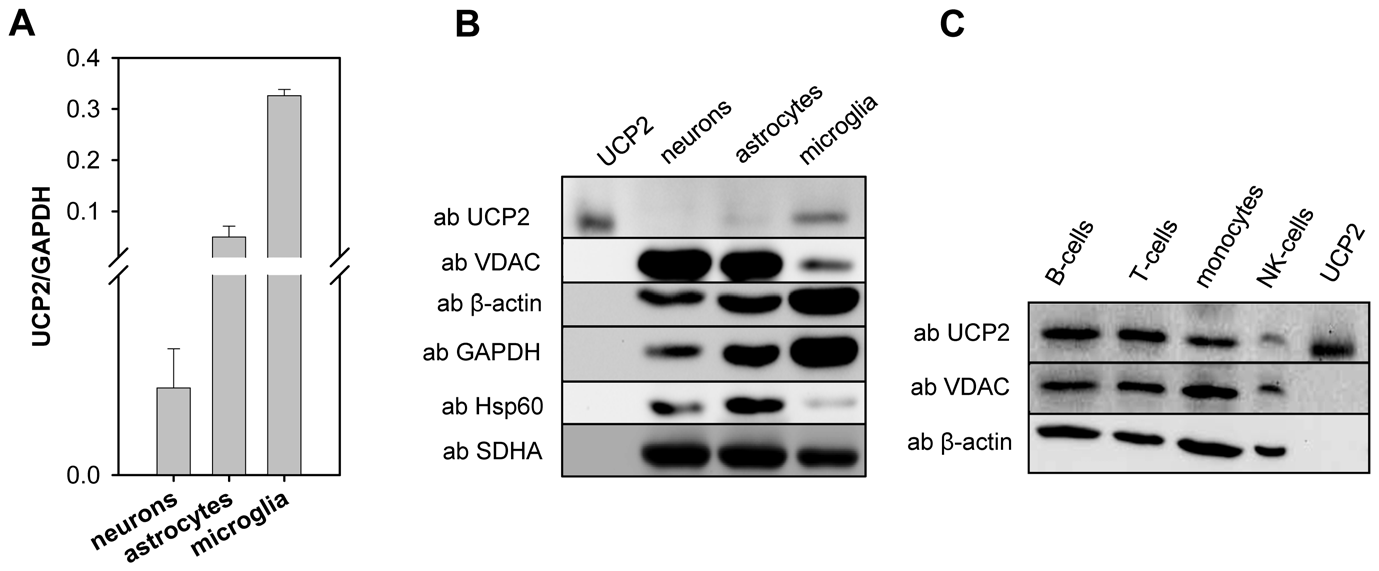
Comparison of UCP2 mRNA (A) and protein content (B) in neurons, astrocytes and microglial cells. (A) mRNA values are presented as ratios of UCP to the house keeping gene GAPDH. Each data point represents the mean value ± SD of three independent cultures each from two litters of independent animals. (B) Western Blot analysis of UCP2 in 20 µg total cellular protein from primary cell cultures of neurons, astrocytes and microglial cells. (C) Protein content in diverse populations of hematopoietic cells. Recombinant human UCP2 was used as a control. 20 µg of total cellular protein was loaded.

To test whether UCP2 can be assigned to microglia, we analysed UCP2 expression in isolated brain cells. Primary cell cultures of neurons, astrocytes and microglial cells were prepared from murine cortex (including hippocampus). Quantitative analysis showed the highest UCP2 mRNA level in microglia (0.326±0.012, Fig. 4A), followed by astrocytes (0.05±0.02) and neurons (0.35±0.16)*10^−3^. The level in microglia can be compared with the expression in thymus, stomach and lungs (Fig. 1). UCP2 mRNA level in neurons is approximately 50 times lower than that of different regions of the brain (Fig. 1, Fig. 3A). Based on this mRNA distribution, we suggest that only microglia express UCP2 protein at detectable levels. Western blot analysis confirmed our suggestion: significant amounts of protein were revealed in microglia cells even by application of 20 µg total protein and to a lesser extent in astrocytes (Fig. 4B). No UCP2 protein was detected in neurons.

### Analysis of UCP2 Protein Expression in Immune Cells

After we have demonstrated the preferential UCP2 expression in lymphatic organs and brain microglia, we analyzed B-cells, T-cells, monocytes and NK-cells (natural killer cells) isolated from blood. Figure 4C reveals a strong protein expression in monocytes, T- and B-cells and weaker expression in NK-cells. This finding supports previous data showing UCP2 expression in B-cells, T-cells, macrophages, dendritic cells and neutrophils [36].

### Time Course of UCP2 Expression After Stimulation of T-cells

**Figure 5:**
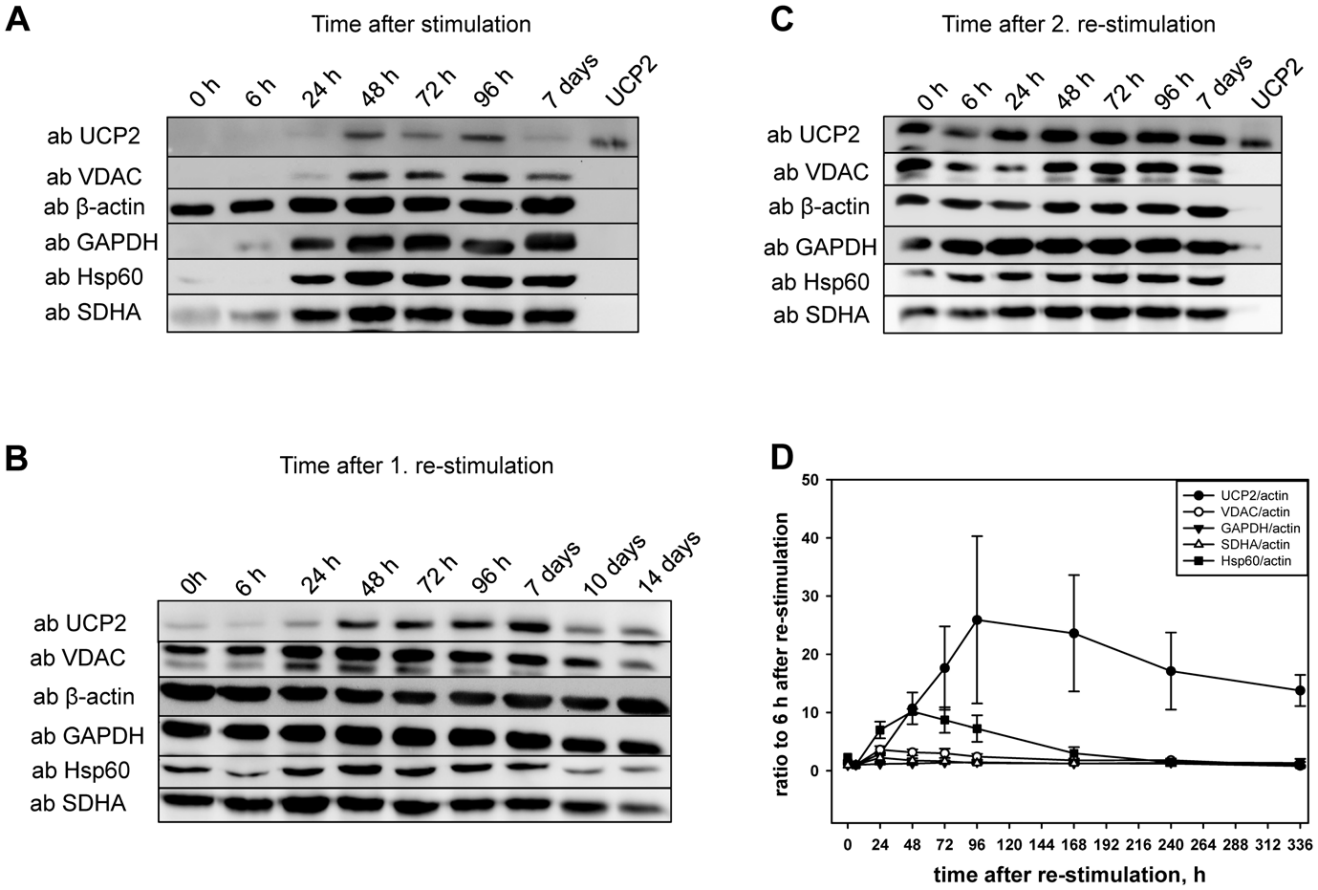
Time course of UCP2 protein expression after unspecific stimulation (A), first re-stimulation (B) and second re-stimulation (C) of isolated T-cells. As mitochondrial markers antibodies against VDAC, Hsp60 and SDHA were used. Cell protein loading was controlled by detection of β-actin and GAPDH. The intensities of UCP2, VDAC, GAPDH, SDHA and Hsp60 were quantified as ratios to the intensity of β-actin. The graph demonstrated the percentage change in expression at 6 hours after the first re-stimulation (D). Each data point represents the mean ± SE of three different WB.

To verify the involvement of UCP2 in immune system function we monitored the protein expression during stimulation of OT-II T-cells. UCP2 expression was analysed by Western Blot at several time points after stimulation up to two weeks (Fig. 5). We detected a significant up-regulation of protein within 24 hours following stimulation (Fig. 5A), reaching a peak at 96 hours (Fig. 5B and 5D). Also the mitochondrial outer membrane protein VDAC, mitochondrial matrix heat shock protein Hsp60 and mitochondrial inner membrane protein SDHA, which we analyzed simultaneously, showed the similar expression patterns. Without re-stimulation, UCP2 expression decreases from day seven after stimulation. The first and second re-stimulation led to a further increase of UCP2 protein expression. Interestingly, whereas simultaneous increase of other mitochondrial proteins (VDAC, Hsp6, SHDA) could be observed during stimulation, only UCP2 is enhanced during re-stimulation (Fig. 5D). The time monitoring of UCP2 expression shows that the modification of protein levels starts several hours after cell activation. Because TCR-mediated activation events occur fast (less than in a few hours) we concluded that UCP2 increase is rather associated with T-cell metabolism alteration and following strong proliferation than with cell activation per se.

We measured similar UCP2 expression kinetics also for CD4 and CD8 T-cells from wild type mice activated with aCD3a/CD28 (Fig. S1A and S1B), confirming that the observed up-regulation of UCP2 is coupled to activation-induced proliferation not only in TCR-transgenic CD4 cells but also in genetically not manipulated C57Bl/6 CD4 and CD8 T cells. The highest UCP2 expression observed in re-stimulation experiment may be explained by the highest proliferative and active state of the T-cells with the maximal increased glycolytic metabolism.

Figure S1C shows that in the presence of IL-2 and in the absence of activation via TCR no significant UCP2 increase was observed, confirming our hypothesis that only cell activation leading to the strong proliferation is associated with UCP2 up-regulation.

### Quantitative Analysis of UCP2 Expression

**Figure 6:**
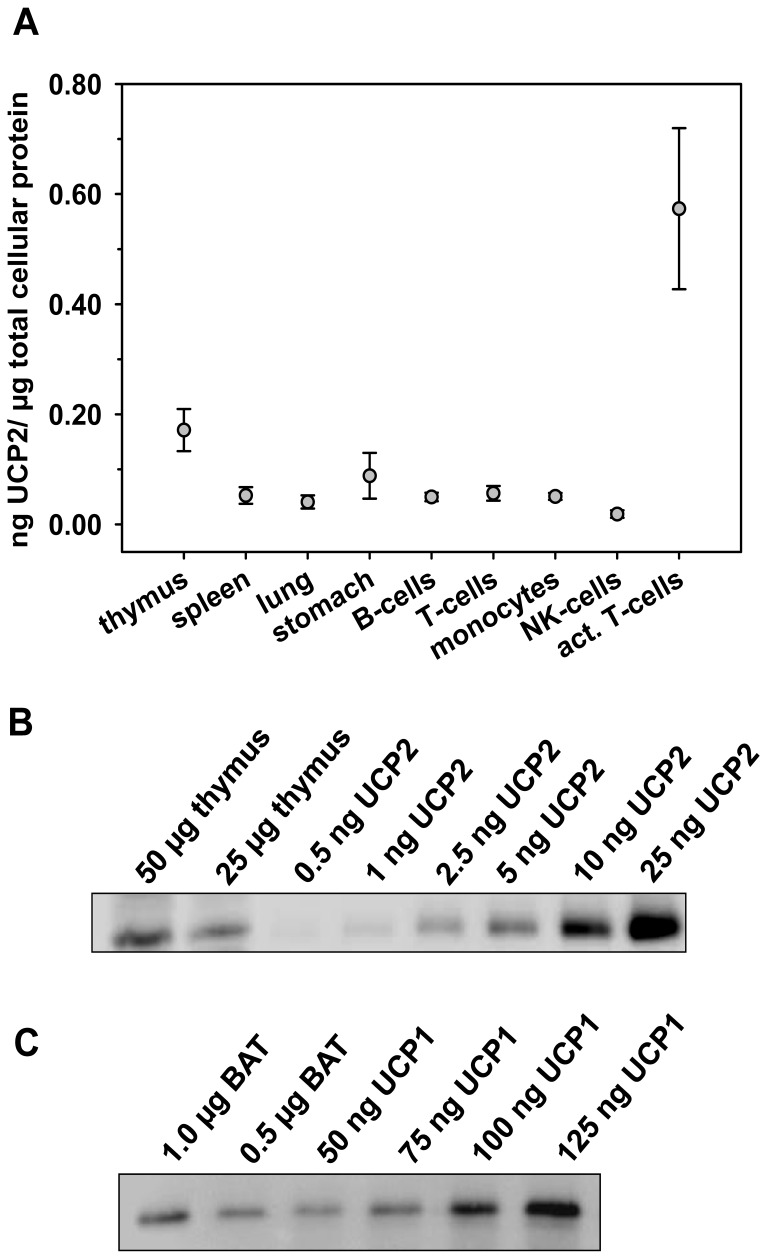
Quantitative analysis of UCP2 expression in tissues using the recombinant proteins UCP2 and UCP1. (A) The comparison of UCP2 amounts in different tissue samples and isolated cells. UCP2 was calculated in ng of µg total protein in whole tissue (cell) lysate. Each data point represents the mean ± SE of six mice. (B) Representative Western Blot showing UCP2 quantification in thymus. (C) Representative Western Blot of UCP1 protein amount compared to the total cell protein in BAT.

The large discrepancy between UCP2 mRNA and protein levels motivated us to estimate the amount of UCP2 in tissues and to compare them with those of UCP1. By loading of defined amounts of recombinant UCP2 and comparing of band intensities between recombinant UCP2 and other tissues (spleen, thymus, stomach and lung) of 12 month old mice we determined the proportion of UCP2 in the whole cell protein extracts. The amount of UCP2 protein in tissues ranges from 0.04 to 0.17 ng/(µg of total cellular protein) (Fig. 6A and 6B). In comparison the amount of UCP2 in different isolated immune cell types was slightly higher at approximately 0.05 ng/(µg of total cellular protein) in B-cells, T-cells and monocytes. NK-cells have only 0.02 ng UCP2 per µg of total cellular protein. Stimulation of T-cells leads to an approximately 10-fold increase in the amount of UCP2 (Fig. 6A). In contrast, we calculated approximately 128,4 ng/(µg of total cellular protein) in BAT taken from 12 month old animals not adapted to cold (Fig. 6C). This means that even in stimulated T-cells the amount of UCP2 is 200 times lower than the level of UCP1 in BAT.

## Discussion

Using quantitative mRNA and Western blot analysis with evaluated antibodies we determined a high UCP2 predominance in mononuclear immune cells of adult mice at both mRNA and protein levels. We confirmed protein expression in B-cells and T-cells and extended the cellular UCP2 expression pattern to monocytes, NK-cells and microglia cells. For the first time we have shown that the stimulation of T-cells leads to an increase of UCP2 in mitochondria during the entire period after cell activation. In contrast to the study of Kizaki et al. on macrophages [39] we extended the period of T-cells investigation to three weeks, including the first stimulation following by two re-stimulations. The time course of UCP2 expression reveals that protein up-regulation follows the stimulation pattern of T-cells, presumably reflecting the enhanced metabolic demand due to rapid lymphocyte proliferation.

It is known that a bioenergetic situation changes dramatically after T-cells become activated (for review [45]). Quiescent T-cells utilize an energy-efficient oxidative metabolism to maintain basic housekeeping but change to glycolysis when stimulated to promote growth, proliferation, migration and effector function in the presence of pathogens [19], [46]. Lymphocytes in a state of high biochemical activity demand a supply of high-energy nucleoside triphosphates for the production of important molecules (cytokines, antibodies, etc.) and the support of enhanced transport and metabolic processes. Through the regulation of the Akt and Myc signalling pathways, uptake of glucose and glutamine is initiated for glycolysis and glutaminolysis respectively [47]. With regard to UCP2, it was shown previously that glutamine abolishes the inhibitory effect of a short upstream open reading frame (uORF, [48]) and induces translation of UCP2 in a concentration-dependent manner [49], [50]. Cytokines are also known to alter the abundance of UCP2 [51]. Therefore the transition of cellular metabolism to glycolysis, leading to an increased glutamine uptake, may simultaneously trigger UCP2 synthesis (for review [52]).

The role of UCP2 during lymphocyte activation is uncertain. However, it becomes more and more obvious that UCP2 is expressed/up-regulated in cells generating ATP mainly by glycolysis and showing a high proliferation rate. Recently it was reported that in human pluripotent stem cells (hPSC) UCP2 may regulate vigour metabolism by facilitating glycolysis via substrate shunting mechanism and preventing glucose oxidation in mitochondria [53]. It was suggested that hPSC require UCP2 repression for full differentiation potential. The comparison of hPSC and lymphocytes, which are fully differentiated, supports the conclusion that UCP2 presence is associated rather with fast proliferation and/or enhanced transport and metabolic processes than with differentiation. This conclusion is in agreement with previous studies in murine embryonic fibroblasts [54] and in bone marrow progenitor cells [43] reporting that the absence of UCP2 or decrease of its level is deleterious for cell proliferation.

UCP2 function at molecular level may be to maintain mitochondrial potential sufficiently low to prevent mitochondrial hyperpolarization [55]. The latter is triggered by T-cell receptor stimulation and is a critical point in decision on T-cell fate [56]. However, in view of the fact that UCP2 is present in the mitochondrial membrane at very low level (Fig. 6), it is presently not understandable whether UCP2 can catalyse a sufficiently high proton leak to change a mitochondrial membrane potential essentially [11]. Another plausible function is a fine-tuning of reactive oxygen species (ROS), which at low levels were described to regulate the expression of specific genes, to trigger signalling pathways and to affect the activities of transcription factors during the increased cell activity (for review [56]). UCP2 was also proposed to regulate mitochondrial glutathione levels rather than regulating ROS directly [26].

The low levels and high experimental variation of UCP2 detected in lungs, intestine and stomach imply that the protein’s presence in these organs may be caused by accumulation of immunocompetent tissues (such as bronchus-associated lymphoid tissue (BALT), alveolar lymph nodes, Peyer’s Patches) and/or by infiltration of activated immune cells due to contact with pathogens, as previously proposed [16].

The most intriguing question is whether UCP2 is present in brain. The detailed analysis of different cell types in the present work shows UCP2 expression in microglia but not in neurons. Because microglial cells are known to act in the immune defence of the central nervous system [57], this finding supports the proposed restriction of protein expression to the immune system. The absence of UCP2 protein expression in neurons under physiological conditions does not exclude the possibility that the protein is expressed under pathological conditions. A protective function for UCP2 has been proposed for a range of diseases, such as ischemia [26], [32], brain trauma [32], epilepsy [58]–[60], Parkinson’s disease [61], diabetes [18], obesity [62], and anorexia nervosa [63]. Interestingly, not only brain degenerative processes, as previously noted [64], but also all other pathophysiological models listed above have a strong immunological background, accompanied by a massive invasion of activated lymphocytes or the activation of phagocytes/microglia [65]. Unfortunately, the proper discrimination of UCP2 expression in different cell types and in restricted areas remains a difficult task, because to date there are no antibodies suitable for the immunohistochemical analysis.

Several research groups have shown that UCP2 mRNA is present only in specific neurons or distinct brain areas ([15], [28], [66]). We cannot exclude the presence of UCP2 in small restricted areas of brain. However, the UCP2 expression in single neurons contradict our presented results and reports from other groups showing that UCP2 abundance is limited to cells, which are able to shift to glycolysis in order to ensure the rapidly increased metabolism during proliferation such as lymphocytes, cancer and embryonic stem cells [53], [67]–[69] Because neurons do not display a high proliferation rate leading to increase of aerobic glycolytic metabolism we suggest that all neurons do not express UCP2 at protein level.

In conclusion, our results support the hypothesis that the function of UCP2 in lymphocytes may be related to the immune challenge state and cell fate decision. Further investigations are needed to analyse whether UCP2 is differently regulated in various modes of T-cell activation and in different types of immune cells. An understanding of UCP2 up-regulation mechanism and function during immune cell activation is essential for defining factors that may control the immune response in inflammatory processes. Thus, UCP2 may represent a potential novel target in diverse inflammatory conditions, such as autoimmune, neurodegenerative and inflammatory diseases.

## Supporting Information

Figure S1 **Time course of UCP2 protein expression in CD4 (A) and CD8 cells (B) after stimulation with abCD3/CD28(A) and CD4 cells without stimulation (C).** Hsp60 and SDHA expression were used as a control for mitochondria amount. GAPDH and β-actin expression were analysed for cellular protein loading. Gels were loaded with 20 µg total cellular protein.
(TIFF)
