# Supplementary figures and images for: Quantification of Uncoupling Protein 2 Reveals Its Main Expression in Immune Cells and Selective Up-Regulation during T-Cell Proliferation
Source: PLoS One. 2012 Aug 3;7(8):e41406. doi: 10.1371/journal.pone.0041406 (PMC3411681; doi:10.1371/journal.pone.0041406)

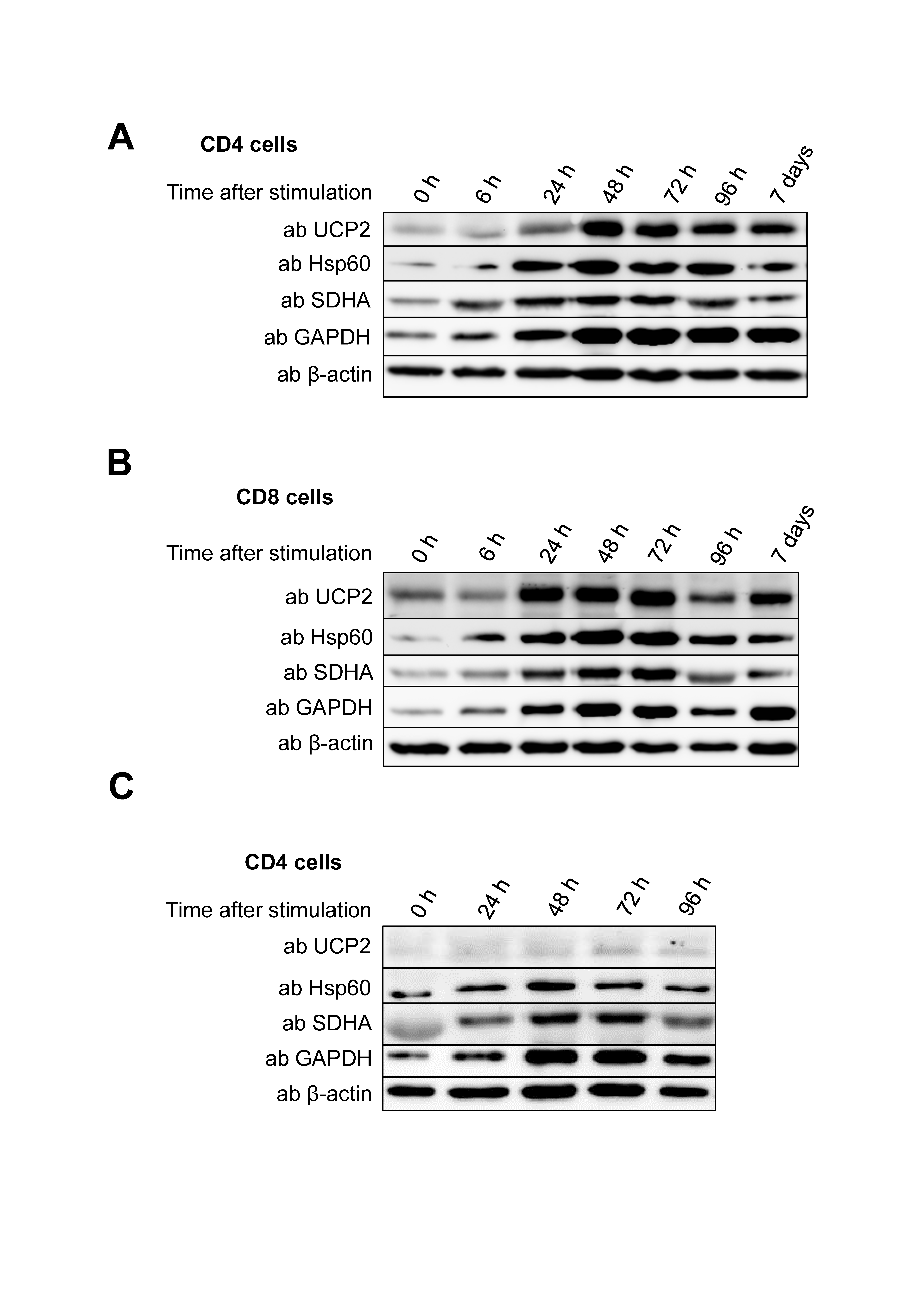

Supplement: Figure S1 — Time course of UCP2 protein expression in CD4 (A) and CD8 cells (B) after stimulation with abCD3/CD28(A) and CD4 cells without stimulation (C). Hsp60 and SDHA expression were used as a control for mitochondria amount. GAPDH and β-actin expression were analysed for cellular protein loading. Gels were loaded with 20 µg total cellular protein. (TIFF) [file pone.0041406.s001.tiff]
